# Validating real-time three-dimensional echocardiography against cardiac magnetic resonance, for the determination of ventricular mass, volume and ejection fraction: a meta-analysis

**DOI:** 10.1007/s00392-023-02204-5

**Published:** 2023-04-20

**Authors:** Thilini Dissabandara, Kelly Lin, Mark Forwood, Jing Sun

**Affiliations:** 1 School of Pharmacy and Medical Science, Griffith University, Gold Coast, Australia; 2 Schools of Medicine and Dentistry, Griffith University, Gold Coast Campus, Gold Coast, QLD 4222 Australia; 3 Menzies Health Institute Queensland, Griffith University, Gold Coast, Australia; 4 Institute for Integrated Intelligence and Systems, Griffith University, Brisbane, Australia

**Keywords:** Cardiac magnetic resonance (CMR), Echocardiography, Left ventricular ejection fraction, Right ventricular ejection fraction, Left ventricular end-systolic volume (LVESV), Left ventricular end-diastolic volume (LVEDV), Left ventricular mass (LVM), Right ventricular end-systolic volume (RVESV), Right ventricular end-diastolic volume (RVEDV)

## Abstract

**Introduction:**

Real-time three-dimensional echocardiography (RT3DE) is currently being developed to overcome the challenges of two-dimensional echocardiography, as it is a much cheaper alternative to the gold standard imaging method, cardiac magnetic resonance (CMR). The aim of this meta-analysis is to validate RT3DE by comparing it to CMR, to ascertain whether it is a practical imaging method for routine clinical use.

**Methods:**

A systematic review and meta-analysis method was used to synthesise the evidence and studies published between 2000 and 2021 were searched using a PRISMA approach. Study outcomes included left ventricular end-systolic volume (LVESV), left ventricular end-diastolic volume (LVEDV), left ventricular ejection fraction (LVEF), left ventricular mass (LVM), right ventricular end-systolic volume (RVESV), right ventricular end-diastolic volume (RVEDV) and right ventricular ejection fraction (RVEF). Subgroup analysis included study quality (high, moderate), disease outcomes (disease, healthy and disease), age group (50 years old and under, over 50 years), imaging plane (biplane, multiplane) and publication year (2010 and earlier, after 2010) to determine whether they explained the heterogeneity and significant difference results generated on RT3DE compared to CMR.

**Results:**

The pooled mean differences for were − 5.064 (95% CI − 10.132, 0.004, *p* > 0.05), 4.654 (95% CI − 4.947, 14.255, *p* > 0.05), − 0.783 (95% CI − 5.630, 4.065, *p* > 0.05, − 0.200 (95% CI − 1.215, 0.815, *p* > 0.05) for LVEF, LVM, RVESV and RVEF, respectively. We found no significant difference between RT3DE and CMR for these variables. Although, there was a significant difference between RT3DE and CMR for LVESV, LVEDV and RVEDV where RT3DE reports a lower value. Subgroup analysis indicated a significant difference between RT3DE and CMR for studies with participants with an average age of over 50 years but no significant difference for those under 50. In addition, a significant difference between RT3DE and CMR was found in studies using only participants with cardiovascular diseases but not in those using a combination of diseased and healthy participants. Furthermore, for the variables LVESV and LVEDV, the multiplane method shows no significant difference between RT3DE and CMR, as opposed to the biplane showing a significant difference. This potentially indicates that increased age, the presence of cardiovascular disease and the biplane analysis method decrease its concordance with CMR.

**Conclusion:**

This meta-analysis indicates promising results for the use of RT3DE, with limited difference to CMR. Although in some cases, RT3DE appears to underestimate volume, ejection fraction and mass when compared to CMR. Further research is required in terms of imaging method and technology to validate RT3DE for routine clinical use.

**Graphical abstract:**

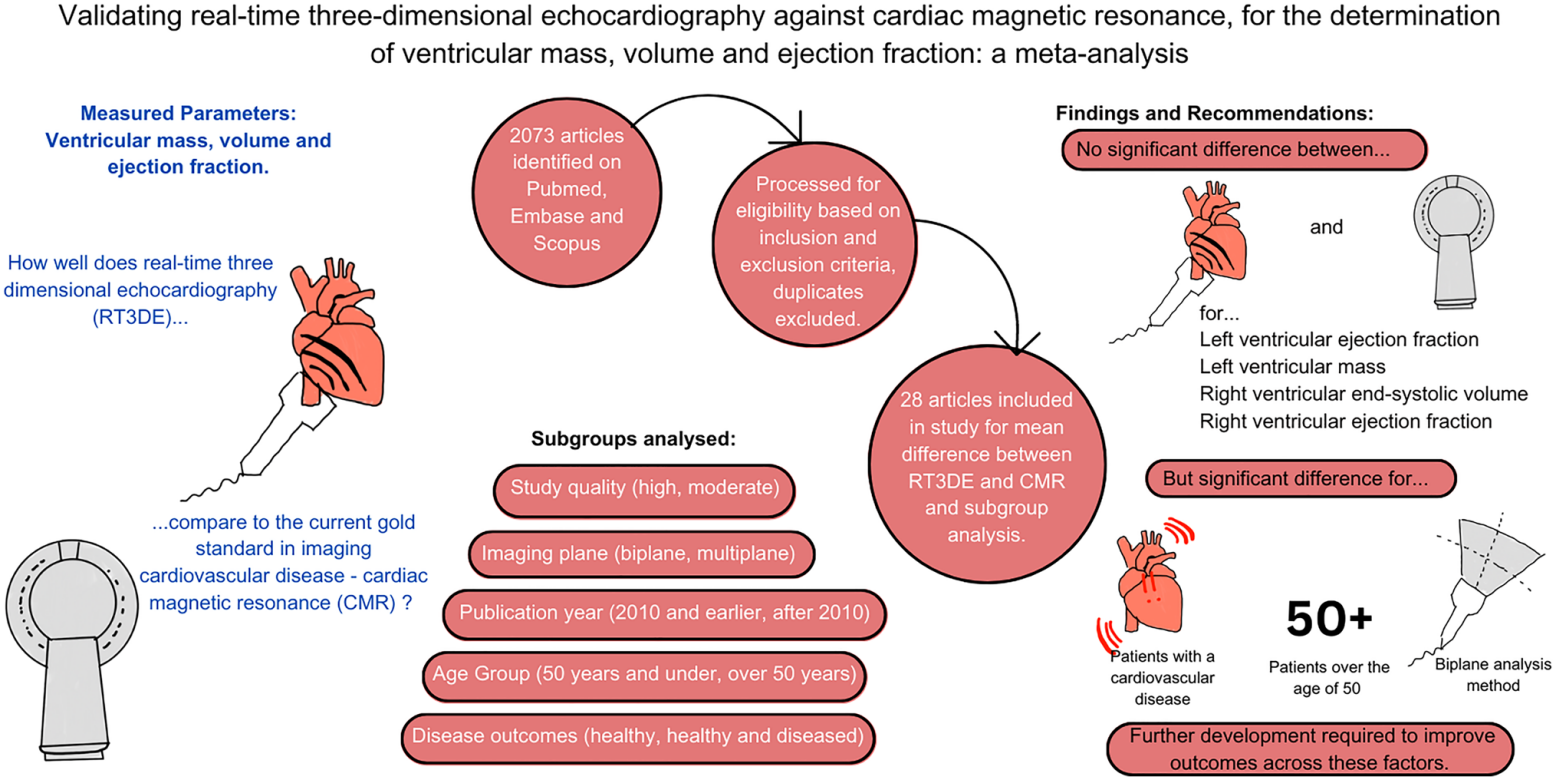

**Supplementary Information:**

The online version contains supplementary material available at 10.1007/s00392-023-02204-5.

## Introduction

Cardiovascular diseases are the leading cause of death worldwide and as such, sensitive diagnostic methods are vital in early diagnosis and, therefore, prevention [1]. The evaluation of ventricular mass, volume and ejection fraction are important parameters in diagnosis [2]. For the last few decades, two-dimensional echocardiography (2DE) has been the routinely used method, with the ability to provide information on each of these parameters [3]. However, two-dimensional echocardiography is limited with regards to the need for geometrical assumptions, foreshortened views and suboptimal endocardial border detection [4]. Two-dimensional echocardiography is operator-dependent, relying on the visual interpretation of moving images, and prone to inter-observer and intra-observer variability and poor test–retest reliability. Moreover, to calculate a volume from 2DE, geometric modelling of chamber shape must be performed and consequently, LVEF estimation from 2DE is subject to bias and error in the presence of pathology. This produces less accurate and less consistent geometric modelling [5].

It has been suggested that 3D echocardiography does not show variability in geometric modelling and has higher inter-observer and intra-observer reliability [6]. RT3DE shows great promise in being included in routine CVD diagnosis in the future, not only because it provides more reliable volume quantification, but also has the ability to crop and visualise specific structures in greater detail. Currently, CMR is considered as the gold standard for three-dimensional imaging that produces the most reliable and accurate imaging of the heart. However, it is both costly and time-consuming in acquisition and processing of images [7].

Previous studies have shown high concordance between RT3DE and CMR in the assessment of ventricular mass volume and ejection fraction [8–12]. This can be attributed to the ability to simultaneously view an image in multiple planes in RT3DE. Chosen long-axis planes can then be analysed using either a biplane or multiplane method for ventricular volumetric analysis and determination of mass. A simpler approach is the biplane method which takes 2-chamber and 4-chamber long-axis views of the image, traces the epicardial and endocardial surfaces of these two planes and uses these to calculate mass and volume. The more complex multiplane approach traces the surfaces of the epicardial and endocardial surfaces of the heart in multiple long-axis planes. This is followed by a correction of the tracings in short-axis views [13].

However, despite the significant advances made in three-dimensional echocardiography, there are still some areas which current studies aim to address, before it is integrated into routine clinical use. The spatial and temporal resolution still do not match that of 2DE, the analysis can be time consuming and the breath-hold time to acquire these images is very long as multiple beats are needed. These beats are stitched together to form a full image which is manually done, potentially causing artefacts in the image. A stitch artefact appears as a fault line in an image, compromising interpretation [14]. Advances are now being made allowing full-volume acquisition to be conducted using only a single beat, instead of multiple to avoid stitch artefacts and shorten breath-hold time [15].

Additionally, to analyse 3DE using either the fully automated or semi-automated algorithms, very high image quality is required. This requires highly trained professionals for image acquisition. Image quality is suggested to be affected not only by the expertise of the professional, but also patient factors. Therefore, current studies aim to programme more sensitive artificial intelligence to analyse images of different quality. They further aim to fully automate the analysis process to speed up the process of RT3DE, to make it a more practical too to be utilised in a clinical setting [7].

This study aims to fill in the research gap by synthesising data from numerous studies which compare RT3DE to the current gold standard, CMR, so a more reliable conclusion can be made surrounding the efficacy of RT3DE, a cheaper and faster tool for CVD diagnosis [7].

## Method

A protocol containing the method and study design is published in Prospero (registration number: CRD42021262783). Studies published from 2000 to present are filtered due to its recent development in RT3DE method, and a broad search is conducted on the databases PubMed, Embase and Scopus with terms (3D echocardiography OR RT3DE OR real-time 3D echocardiography) AND (cardiac magnetic resonance OR CMR). Total articles are noted and then abstract and title screening is conducted to ensure studies fit within inclusion and exclusion criteria based on PICO approach. P: Live human adults (18+ years) of all ethnicities and both genders are the target population of the study, excluding children. I: The studies must mention terms RT3DE. Imaging conducted at rest is taken, as results during stress echocardiography can be significantly different. Post-mortem analyses and computer simulation which can produce significantly different results are excluded. C: CMR to be included. O: outcomes including at least one of the following primary outcomes: LVESV, LVEDV, LVEF, LVM, RVESV, RVEDV and RVEF.

All records are collected onto an Endnote library and then full-text articles for each included study are found. After screening the full-text articles, studies are eliminated if data are not present on the primary outcomes of the meta-analysis and those with duplicated or overlapping data. The Endnote library is then compressed and sent to a peer, along with the search terms, search databases and eligibility criteria, to mitigate reviewer bias. The final screening is conducted by a third reviewer and a final decision is made on the articles to be used for data extraction.

Data extraction is conducted on a Microsoft Excel document with each primary outcome on a different sheet. Data are extracted for primary outcomes (LVESV, LVEDV, LVEF, LVM, RVESV, RVEDV and RVEF) and subgroups. Subgroups of continuous variables from studies are converted to categorical variables to prepare for subgroup analysis. The categories used in this study include age (1 = 50 and less, 2 = more than 50 years old), disease condition (1 = disease, 2 = disease and healthy, 3 = healthy), quality of study (1 = high and 2 = medium), publication year (1 = 2010 and earlier, 2 = after 2010) and RT3DE analysis planes (1 = biplane, 2 = multiplane).

Study characteristics including study location, gender ratio, average age, study design, imaging method and brand, analysis method, statistics, disease conditions and outcome measures are all collected. The data collected for analysis include RT3DE and CMR mean values, standard deviations and sample size for calculation of effect size and mean difference. Additionally, regression values and Bland–Altman test values are also collected. Where mean and standard deviation are not reported, median values are taken to equal the mean and standard deviation is taken to be range/4. All data are checked by two independent reviewers to reduce human error and bias.

The quality assessment check is conducted using the The Grading of Recommendations Assessment, Development and Evaluation (GRADE) tool where only high- or medium-quality studies are used [16]. Two authors independently reviewed studies using this approach to ensure study quality is reliably noted.

All data analysis is conducted on STATA SE version 17. Egger regression analysis was used to assess publication bias and if the *p* value is less than 0.05, this indicates publication bias. If publication bias is identified, further sensitivity analysis was used to assess the publication bias is due to a single study and further removal of the study was conducted to validate the final results.

A random model using effect size measures including standardised mean difference and effect size (using Cohen’s *d* method) between RT3DE and CMR will be used for all continuous variables including primary outcomes in ventricular volumes, mass, ejection fraction. Mean difference is used to indicate whether there is any significant difference between RT3DE and CMR. Effect size is used to indicate the size of the difference between CMR and RT3DE. Additionally, concordance results and Bland–Altman analysis results are pooled using mean and recorded. Many studies report standard deviation instead of upper and low limits of agreement (LOA), which is required for this study. In this case, the standard deviation is converted to LOAs using the formulas [17]:

$$\mathrm{upper}\, \mathrm{LOA}=\mathrm{bias}+1.96\times \mathrm{standard}\, \mathrm{deviation},$$

$$\mathrm{lower}\, \mathrm{LOA}=\mathrm{bias}-1.96\times \mathrm{standard}\, \mathrm{deviation}.$$

Heterogeneity analysis using *I*^2^ will be used to identify the level of variability across studies. An *I*^2^ value of more than 50% indicates a high level of heterogeneity. Subgroup analysis was conducted to identify sources of heterogeneity if *I*^2^ is more than 50%.

All data and selected studies were checked by two researchers to ensure no errors in data collection were made which can lead to erroneous conclusions. Inclusion and exclusion criteria are predetermined and applied uniformly to all studies to ensure objective selection of studies. Statistical analyses were conducted to consider the possibility of publication bias. As this is a meta-analysis, any human ethical considerations were not required for this study.

## Results

**Fig. 1:**
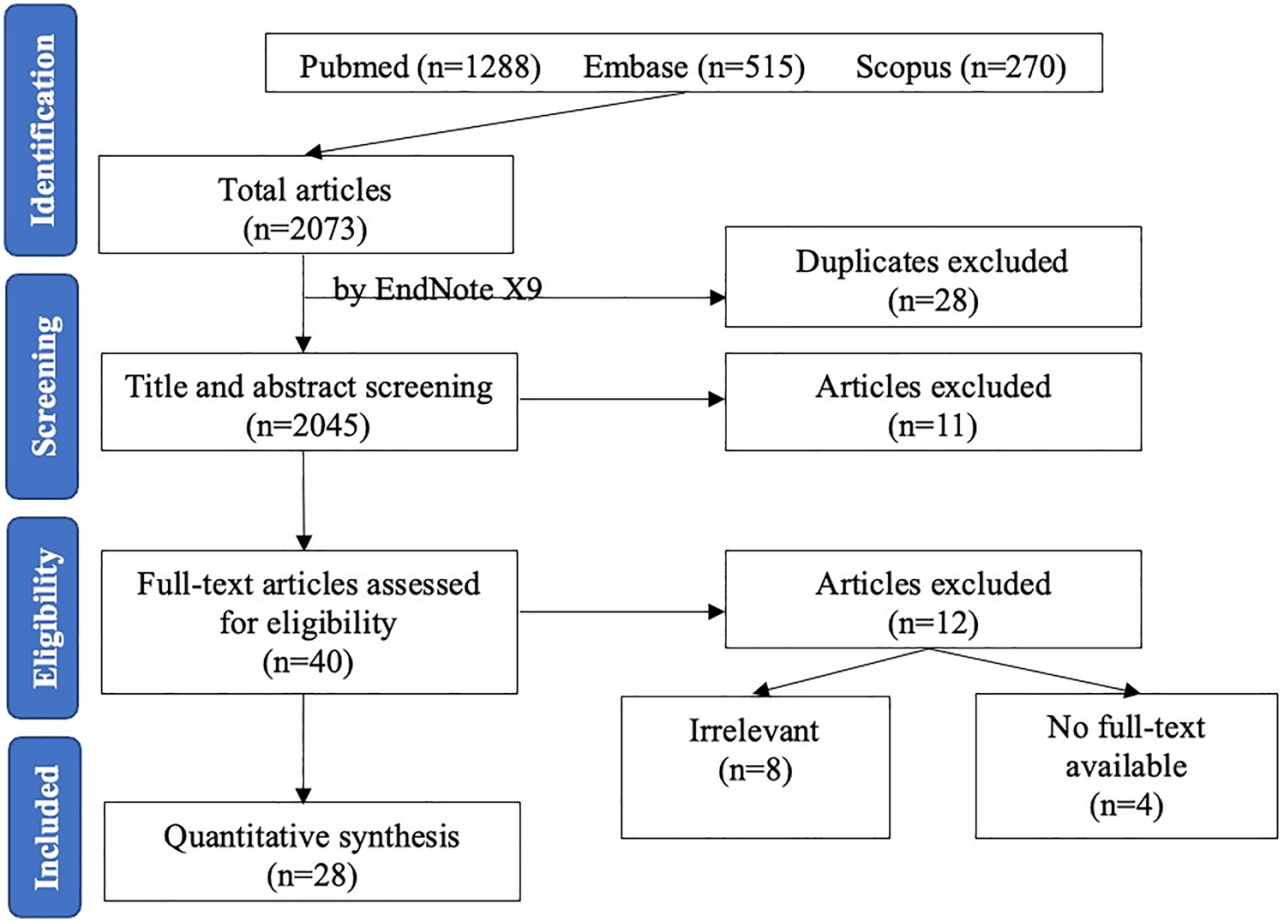
PRISMA flow chart of study selection

The search process can be seen on Fig. 1 and included studies and study characteristics on Table 1. A total of 2073 potential studies from the databases Pubmed, Embase and Scopus were identified. After the removal of 28 duplicates, 40 articles remained. Full text screening was conducted on the remaining articles and 12 were excluded due to irrelevance or data insufficiency. Overall, 28 articles are used for quantitative synthesis in this meta-analysis.

**Table 1:** Study characteristics for all included studies

Study	Location	Male/female	Average age	Imaging methods	Analysis method	RT3DE analysis planes	Analysis	RT3DE beat number	Statistics	Disease conditions	Outcomes measures	Quality	Interval between RT3DE and CMR
Avegliano, Costabel [17]	Argentina	35/13	57.4	RT3DE (matrix-array transducer X4, Phillips iE33) CMR (SIGMA-CVi1.5)	RT3DE (QLAB 8.0, Philips medical systems) CMR (MASS, MEDIS Medical Imaging Systems)	Biplane	Semi-automatic, manual tracing using modified simpsons rule	4 beat	Students *t* test, Chi square test, Lin method for concordance, Bland–Altman analysis for bias	Ventricular hypertrophy (nonobstructive septal hypertrophy, obstructive septal hypertrophy, apical hypertrophy)	LVM	High	Within 7 days
Bech-Hanssen, Polte [18]	Sweden	70/13	56.5	RT3DE (Vivid E9, GE Healthcare, Milwaukee, WI, 3D matrix-array transducer) CMR (Achieva, Philips Healthcare, five-channel phased-array cardiac coil)	RT3DE [EchoPAC (4DLVQ, GE Healthcare)] CMR [ViewForum (Philips Healthcare)]	biplane	Semi-automatic, manual tracing using modified simpsons rule	4–6 beats	Pearson correlation coefficient for concordance, paired students *t *test, Wilcoxon rank test, Friedmans test	Aortic regurgitation, mitral regurgitation	LVESV, LVEDV, LVEF	High	Within 4 h
Bicudo, Tsutsui [19]	Brazil		32	RT3DE (X4 Matrix-array transducer, SONOS 7500, Philips Medical Systems) CMR (Sigma CV/i, General Electric Medical Systems)	RT3DE [Q-Lab versions 4.0 and 4.2.1 (Philips Medical Systems)] CMR [Report Card (General Electric Medical Systems)]	Multiplane	Semi-automatic, manual tracing using modified simpsons rule	4 beat	Students *t* test, Chi square test, Lins agreement for concordance, Bland–Altman for bias, inter-observer and intra-observer variability	Hypertrophic cardiomyopathy	LVESV, LVEDV, LVEF, LVM	Moderate	93 ± 47 days
Caiani, Corsi [20]	USA	13/8	48	RT3DE [matrix-array transducer (X 4)] CMR [1.5 T scanner (General Electric)]	RT3DE (not specified) CMR (MASS Analysis, General Electric)	Multiplane	Automatic, manual tracing using modified simpsons rule	4 beat	Paired *t* test, Pearson correlation, SEE, Bland–Altman for bias	Coronary artery disease, dilated cardiomyopathy, myocardial infarction, aortic disease, right atrial mass, mitral valve regurgitation	LVM	Moderate	Within 24 h
Chang, Kim [28]	South Korea	58/11	58.2	RT3DE [Acuson SC2000; Siemens Medical Solutions, acquisition transducer (4Z1c)] CMR [1.5-Tscanner (Magnetom Avanto, syngo MR; Siemens Healthcare)]	RT3DE [(Volume Cardiac Analysis Package—Volume Left Ventricular Analysis version 1.6) on the SC2000 system] CMR (Argus version 4.02; Siemens Healthcare)	Multiplane	Automatic contouring algorithm with manual adjustments for endocardial border, manual tracing of epicardial border, automated analysis		Bland–Altman for bias, intraclass correlation coefficient for concordance	Hypertrophic cardiomyopathy	LVM	High	Within 24 h
Driessen, Kort [29]	Netherlands	19/16	49.7	RT3DE [Philips-iE33 with X5–1 5 MHz transducer (Philips Medical Systems)] CMR [1.5-Tesla or Philips Ingenia 1.5-Tesla scanner (Philips Medical Systems)]	RT3DE [Qlab version 8.1 (Philips Medical Systems)], CMR [Philips Cardiac Explorer (Philips EWS (release 2.6)]		Semi-automatic, border detection with manual adjustments		Students *t* test, Bland–Altman analysis, ICC for concordance	Hypertrophic cardiomyopathy, dilated cardiomyopathy, cardiomyopathy, pericarditis, myocarditis, ischaemic heart disease	LVESV, LVEDV, LVEF	Moderate	Within 8 h
Gopal, Chukwu [22]	USA	35/36	56	RT3DE [matrix array transducer (2 × 4 MHz) (X4, Philips Imaging Systems)] CMR (1.5-T scanner (Sonata, Siemens)	RT3DE (TomTec Imaging Systems GmbH) CMR (Medis Mass)	Multiplane	Semi-automatic	4 beat	Pearsons correlation, linear regression, Bland–Altman analysis	Normal	RVESV, RVEDV, RVEF	High	
Grapsa, O'Regan [31]	UK	17/63	42.8	RT3DE (GE Vivid 7 scanner (Horton Norway) equipped with a central X4 transducer) CMR (1.5 T Philips Achieva system)	RT3DE (4D analysis, TomTec) CMR [View Forum software (Philips)]		Semi-automatic	2.5 beat	Students *t* test, intraclass correlation for concordance, Bland–Altman analysis	Pulmonary arterial hypertension	RVESV, RVEDV, RVEF	Moderate	Within 2 h
Jaochim Nesser, Sugeng [23]	USA	17/14	60	RT3DE [SONOS 7500, Philips Medical Systems, Andover, Massachusetts, USA equipped with a fully sampled matrix-array transducer (64)] CMR (1.5-T scanner (Sonata, Siemens)	Both analysed with 4D-LV Analysis software (TomTec Imaging Systems)		Semi-automatic	4 beat	Paired *t* test, Pearson correlation coefficient, Bland–Altman for bias	Normal, coronary artery disease, dilated cardiomyopathy, apical hypertrophic cardiomyopathy	LVESV, LVEDV, LVEF	Moderate	Within 24 h
Jenkins, Bricknell [45]	Australia	41/9	64	RT3DE [matrix-array ultrasonographic transducer (X4 transducer, Philips Sonos 7500 system, Andover, Massachusetts)] CMR [Sonata 1.5-T scanner (Siemens)]	RT3DE (4D analysis, Tomtec Gmbh) CMR [Cardiac Image Modelling software (CIM version 4.2)]	Multiplane	Semi-automatic border detection	4 beat	Pearson correlation coefficient, Bland–Altman analysis	Normal, regional wall motion abnormality, hypertension	LVESV, LVEDV, LVEF, LVM	High	Within 1 h
Kim, Cohen [24]	USA	21/6	61	RT3DE (iE33 echocardiographic system (Philips Medical Systems, X3-1 matrix-array transducer) CMR (1.5-T MRI Siemens Symphony scanner)	RT3DE (TomTec Imaging Systems) CMR (Argus; Siemens Medical Systems)	Multiplane	Semi-automatic with manual tracing and automatic detection	3–5 beats	Linear regression with Pearson correlation coefficient, Bland–Altman analysis	Ischaemic cardiomyopathy, non-ischaemic cardiomyopathy, left bundle branch block, right bundle branch block, myocardial infarction	RVESV, RVEDV, RVEF	Moderate	7.3 ± 11.9 days
Kuhl, Schreckenberg [36]	Germany			RT3DE (Sonos 7500, Philips, X4, Philips) CMR (1.5-T scanner (Intera, Philips)	RT3DE (CardioView RT, TomTec) CMR (manual)	Multiplane	Semi-automatic with manual tracing and automatic border detection and volume quantification	4 beats	Pearson correlation, Bland–Altman analysis	Normal, dilated cardiomyopathy, ischaemic cardiomyopathy	LVESV, LVEDV, LVEF	Moderate	Within 24 h
Leibundgut, Rohner [37]	Switzerland	70/18	50	RT3DE (Philips iE33 ultrasound system equipped with a matrix-array X3-1 transducer) CMR [1.5-T magnet (Mag- netom Avanto or Espree; Siemens Medical Solutions)]	RT3DE (4D RV-Function CAP 1.1; TomTec Imaging Systems) CMR (Argus; Siemens Medical Solutions)	Multiplane	Automatic with manual correction	7 beats	Wilcoxon signed-rank test, intraclass correlation coefficient, Bland–Altman analysis	Other cardiomyopathies, pericarditis, myocarditis	RVESV, RVEDV, RVEF	High	Within 24 h (for 97% patients)
Li, Wang [38]	China	10/13	51.6	RT3DE [X3-1(iE33, Philips Health care) and a 4Z1C matrix array transducer (Siemens Acuson SC2000)] CMR [3.0 Tesla magnetic resonance scanner (TimTrio; Siemens)]	RT3DE (TomTec Imaging Systems) CMR (Argus; Siemens Medical Systems)		Semi-automatic with manual tracing	4–7 beats	Pearson correlation coefficient, Bland–Altman analysis	Pulmonary hypertension	RVESV, RVEDV, RVEF	Moderate	Within 24 h
Lu, Chen [34]	Australia	36/24	45	RT3DE [GE Vivid 9 V3 4 V transducer (frequency of 1.7/3.3 MHz)] CMR [1.5 Tesla MRI system (Siemens Avanto, Siemens Medical Solutions)]	RT3DE (TomTec Imaging Systems) CMR (Argus; Siemens Medical Systems)		Semi-automatic with manual tracing	4 beats	Pearson correlation coefficient, Bland–Altman analysis	Cardiomyopathy, ventricular arrhythmias, congenital heart disease, ischaemic heart disease, perimyocarditis	RVESV, RVEDV, RVEF	High	
Macron, Lim [39]	France	34/16	59	RT3DE (3D matrix-array transducer [Vivid E9 scanner, 3 V-D probe (2.5 MHz)] CMR (1.5-T system (Avanto, Siemens Medical Systems, Erlangen, Germany), cardiac 6-element phased-array coil)	RT3DE (Echo PAC-PC, GE Healthcare) CMR (Argus, Siemens Medical Systems)	Multiplane	Automatic with manual correction	1, 2 and 4 beats	Pearson correlation coefficient, Bland–Altman analysis, *t *test	Coronary artery disease, valvular heart disease, dilated cardiomyopathy, hypertrophic cardiomyopathy	LVESV, LVEDV, LVEF	High	Within 24 h
Marsan, Westenberg [30]	Netherlands	37/15	62	RT3DE (iE33; Philips Medical Systems equipped with an X3, fully sampled matrix transducer) CMR [1.5 T scanner equipped with powertrack 6000 gradients (Gyroscan ACS-NT/Intera; Philips Medical Systems)]	RT3DE (Q-Lab Version 6.0; Philips Medical Systems) CMR [MASS analytical software (Medis)]	Multiplane	Semi-automatic		Students *t* test, Pearson correlation, Bland–Altman analysis	Ischaemic cardiomyopathy and left ventricular aneurism	LVESV, LVEDV, LVEF	High	Within 24 h
Miller, Pearce [32]	UK	41/19	61	RT3DE [iE33; Philips Healthcare, equipped with a 3D matrix-array transducer (X3-1)] CMR [1.5-Tesla scanner (Avanto; Siemens Medical Imaging)]	RT3DE [QLAB (3DQ Advanced, Philips)] CMR [CMRtools (Cardiovascular Imaging Solutions)]		Semi-automatic	4 beats	Wilcoxon rank, spearman correlation, Bland–Altman analysis	Ischaemic heart disease, normal, cardiomyopathy, valvular heart disease, pericardial disease, ascending aortic aneurysm, intra-cardiac mass	LVESV, LVEDV, LVEF	High	Immediately after
Moceri, Doyen [40]	France	17/7	58	RT3DE (X3-1 transducer, Philips iE33 xMA- TRIX echocardiography system, Philips) CMR (1.5-T scan- ner with a phased-array torso coil)	RT3DE (Xcelera workstation – 3DQ Advanced quantification tool) CMR (manual)		Semi-automatic border detection	4 beats	*z* statistic for correlation, Bland–Altman analysis	Ischaemic cardiomyopathy, dilated cardiomyopathy, heart failure with preserved ejection fraction	LVESV, LVEDV, LVEF	Moderate	Within 24 h
Mor-Avi, Sugeng [25]	USA	13/8	48	RT3DE [SONOS 7500, Philips, matrix array transducer (X4, 2–4 MHz)] CMR (1.5-T scanner (General Electric) with a phased-array torso coil)	RT3DE (3DQ-QLab, Philips) CMR (MASS Analysis, General Electric)	Biplane	Semi-automatic with manual tracing	4 beats	Paired *t *test, Pearson correlation coefficient, Bland–Altman for bias	Coronary artery disease, dilated cardiomyopathy, myocardial infarction, aortic abnormalities, right atrial mass, mitral valve disorder	LVM	Moderate	Within 24 h
Oe, Hozumi [41]	Japan	17/4	54	RT3DE [Sonos 7500 system (Philips Medical Systems) using second harmonic imaging with a matrix array X4 transducer (2–4 MHz)] CMR [1.5-T whole-body scanner (Magnetom Vision, Siemens Medical Systems)]	RT3DE [4D Cardio-View (TomTec Imaging Systems)] CMR (ARGAS, Siemens Medical Systems)	Multiplane	Semi-automatic with manual tracing	4 beats	Regression analysis, Bland–Altman analysis	Hypertrophic cardiomyopathy, hypertensive heart disease	LVM	Moderate	
Qi, Cogar [43]	Taiwan	40/18	58.97	RT3DE (iE33, Philips Medical Systems) equipped with a 4X matrix-array transducer) CMR (1.5 T scanner (Magnetom Sym- phony, Siemens) using a body-array coil for signal detection)	RT3DE (TomTec Echoview version 5.2) CMR (ARGUS, Siemens Medical System)	Multiplane	Semi-automatic with manual tracing	4 beats	Pearson correlation coefficient, Bland–Altman analysis	Coronary artery disease, normal, dilated cardiomyopathy, atrial septal defect, ventricular septal defect, valvular heart disease	LVESV, LVEDV, LVEF, LVM	High	Within 24 h
Shibayama, Watanabe [42]	Japan	30/11	63	RT3DE (ACUSON SC2000 volume imaging ultrasound system (Siemens Medical Solutions) with a 4Z1c volume imaging transducer CMR (Magnetom Sonata 1.5- T MR scanner (Siemens Medical Solutions) using a 6-channel phased-array body and spine coil	RT3DE [off-line Syngo® SC2000 Workplace (eSie LVA, Siemens AG)] CMR [Argus Function VA30 (Siemens Medical Solutions)]	Biplane	Automatic with manual correction and semi-automatic	1 beat	Pearson correlation coefficient, Bland–Altman analysis, Wilcoxon rank-signed	Normal, valvular heart disease, dilated cardiomyopathy, hypertrophic cardiomyopathy, myocardial infarction, coronary artery disease, wall motion abnormalities	LVESV, LVEDV, LVEF	Moderate	Within 6 h
Squeri, Censi [44]	Italy	42/24		RT3DE (Philips iE33 scanner with matrix-array ultrasonographic transducer (X3.1 transducer; Philips Medical Systems) CMR (.5-T Philips scanner (Achieva; Philips Medical Systems) standard body coil 5-channel phased-array cardiac coil	RT3DE [Qlab software (Version 6.0; Philips Medical Systems)] CMR (Philips ViewForum; Philips Medical Systems)		Automatic with manual correction		Pearson correlation coefficient, Bland–Altman analysis, paired *t *test	Arrhythmogenic right ventricular dysplasia, hypertrophic cardiopmyopathy, dilated cardiomyopathy, myocarditis, acute coronary syndrome	LVESV, LVEDV, LVEF	High	Within 2 days
Sugeng, Mor-Avi [26]	USA, Austria, Germany	19/9	53	RT3DE (iE33 imaging system (Philips) with matrix-array transducer (X3-1) CMR (1.5-T scanner (Siemens) with a phased-array cardiac coil)	Not specified	Multiplane	Automatic with manual correction	4 beat	Linear regression analysis, Bland–Altman analysis	Congestive heart failure, secondary pulmonary hypertension, primary arterial hypertension, congenital heart disease, coronary artery disease	LVESV, LVEDV, LVEF	Moderate	Within 24 h
Sugeng, Mor-Avi [27]	USA, Austria, Germany	17/14	60	RT3DE [SONOS 7500 scanner (Philips) equipped with matrix-array transducer (X4)] CMR (1.5-T Sonata scanner (Siemens) with phased-array cardiac coil)	RT3DE [4D-LV Analysis software (TomTec Imaging Systems)] CMR [prototype software (TomTec)]	Multiplane	Semi-automatic	4 beat	Pearson correlation coefficient, Bland–Altman analysis, paired *t *test	Normal, coronary artery disease, dilated ventricles, apical hypertrophic cardiomyopathy	RVESV, RVEDV, RVEF	Moderate	Within 24 h
Yap, van Geuns [13]	Netherlands	13/5	30	RT3DE [matrix-array transducer (X4, 2–4 MHz) connected to a commercial ultrasound system (SONOS 7500, Philips Medical Systems)] CMR [1.5-T MRI scanner with four- element phased-array receiver coil (Signa CV/I, GE Medical Systems)]	RT3DE [3DQ-Qlab (Philips Medical Systems)] CMR (CAAS-MRV, Pie Medical Imaging)	Biplane and multiplane	Semi-automatic	4 beat	Paired *t *test, Pearson correlation coefficient, Bland–Altman for bias	Concentric left ventricular hypertrophy	LVM	Moderate	Within 26 ± 14 days
Zhang, Sun [35]	Australia, China, Hong Kong	38/21	62	RT3DE (special transducer (4Z1c) that has a matrix array, Acuson SC2000 system; Siemens Medical Solutions) CMR (1.5 T magnet (Signa Infinity Twin Speed with Excite Technology; General Electric Medical Systems), phase array coil)	RT3DE (TomTec v1.2, TomTec Imaging Systems on Acuson SC2000 system) CMR [GE ADW4.1 workstation (the ARGUS software mass analysis)]	Multiplane	Automatic with manual correction	1 beat	Linear regression, intraclass correlation, Bland–Altman	Normal, hypertension, pulmonary heart disease, coronary heart disease	RVESV, RVEDV, RVEF	High	Within 24 h

The majority of mean effect estimates of individual studies for LVESV, LVEDV, LVEF, RVESV, RVEDV and RVEF, as reflected by the overall effect estimate, lie to the left of the central line, favouring RT3DE over CMR. Although for LVM, the majority of mean effect estimates, as reflected by the overall effect estimate, lie to the right of the central line, favouring CMR over RT3DE.

The 28 studies included were published between the years 2004 and 2017 and were all case–control studies. The total 1215 (800 males, 415 females) patients received RT3DE followed by CMR across all studies and all participants received both RT3DE (case) and CMR (control) imaging. Exceptions include Caiani et al. [21] where two patients received only CMR as they had dilated cardiomyopathy preventing their heart from being able to fit into the pyramidal scan volume for RT3DE and Zhang et al. [35] where two patients did not have adequate image quality for RT3DE analysis. Participants were recruited from 17 different countries including Argentina [18], Sweden [19], Brazil [20], USA [21–27], South Korea [28], Netherlands [13, 29, 30], UK [31, 32], Australia [33–35], Germany [26, 27, 36], Switzerland [37], China [35, 38], France [39, 40], Japan [41, 42], Taiwan [43], Italy [44], Austria [26, 27] and Hong Kong [35].

**Fig. 2:**
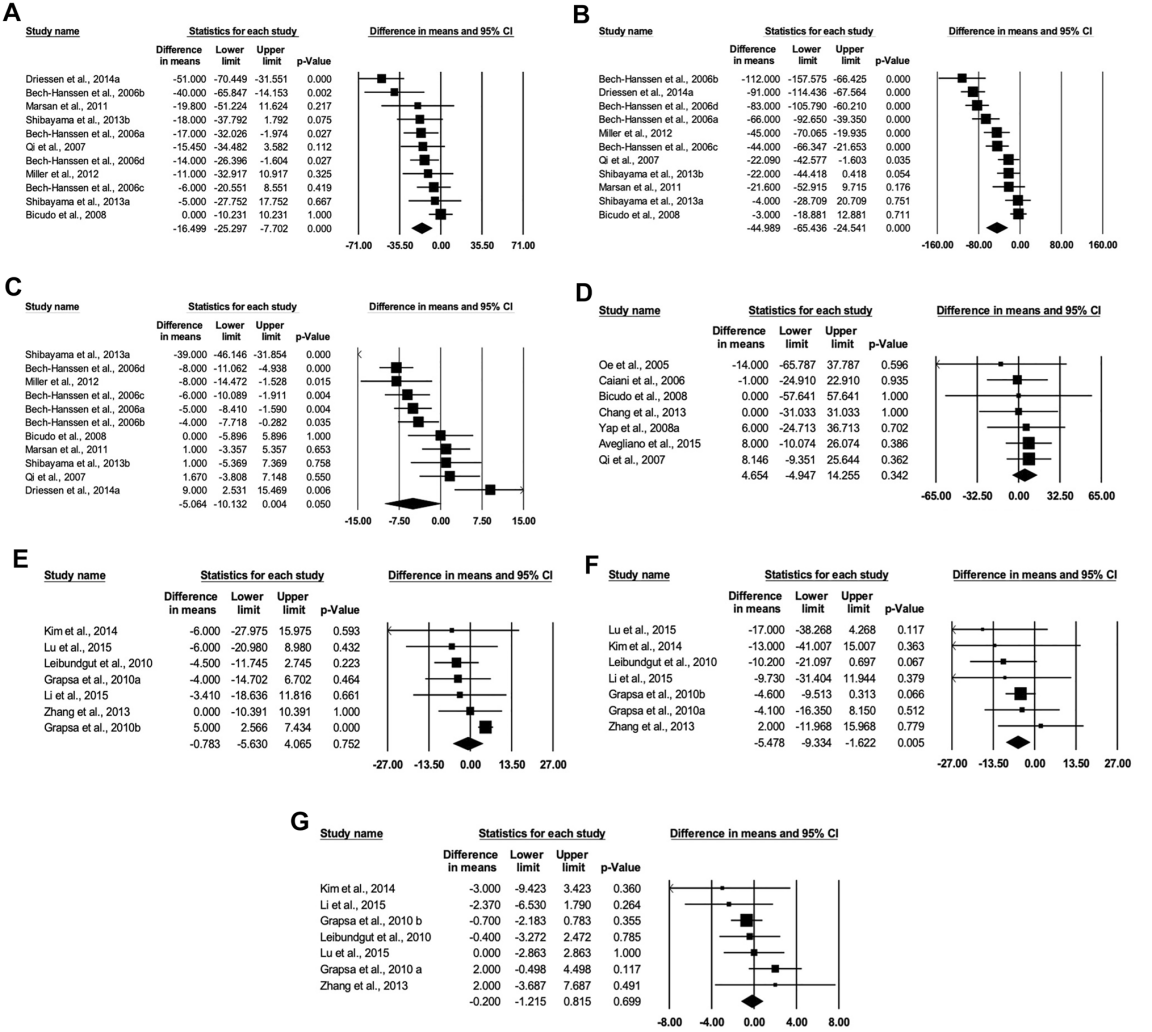
Forest plots for RT3DE assessment of LVESV (**A**), LVEDV (**B**), LVEF (**C**), LVM (**D**), RVESV (**E**), RVEDV (**F**) and RVEF (**G**) compared to CMR

All studies include had at least one of the required outcome variables including LVESV, LVEDV, LVEF, LVM, RVESV, RVEDV and RVEF. It should be mentioned here that insufficient studies were available on the right ventricular mass (RVM) outcome variable and, therefore, this variable was not included in the meta-analysis. Information on the RT3DE and CMR imaging technologies, analysis method (automatic or semi-automatic), beat number used for RT3DE imaging, disease conditions of patients, age group, male to female ratio and RT3DE analysis plane (biplane or multiplane) were collected as these varied among the 28 studies, summarised in Table 1. In addition key results from the GRADE quality assessment as well as statistical analysis tests used in each study are also summarised in Table 1 (Fig. 2).

**Fig. 3:**
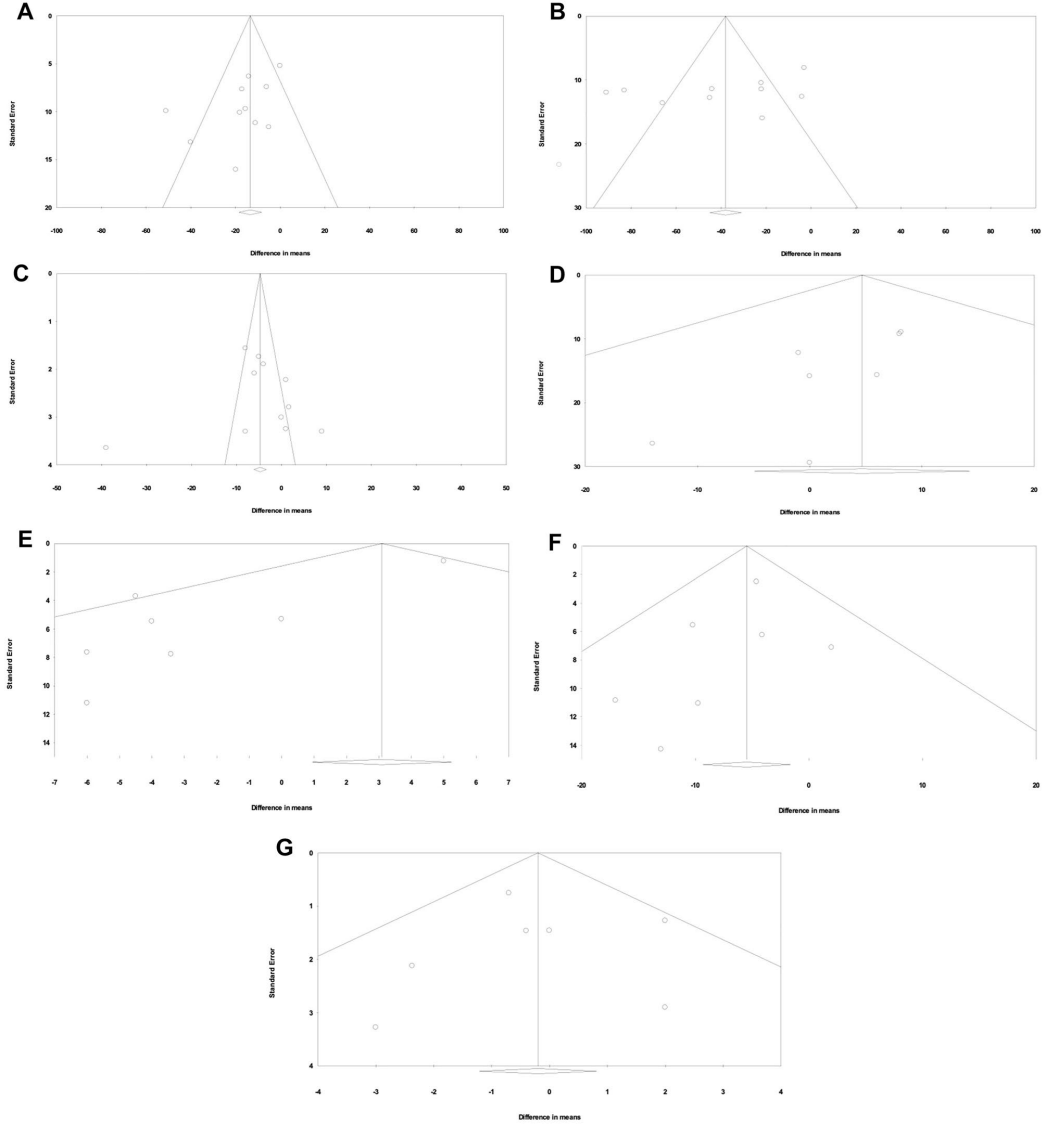
Funnel plots for RT3DE assessment of LVESV (**A**), LVEDV (**B**), LVEF (**C**), LVM (**D**), RVESV (**E**), RVEDV (**F**) and RVEF (**G**) compared to CMR]

The studies used in this meta-analysis were either moderate or high quality based on the GRADE assessment conducted. Only moderate or high-quality studies were used in an attempt to prevent bias. The results from the Egger regression test for publication bias displayed in STable 5 indicated no significant bias for LVESV, LVEDV, LVEF, LVM, RVEDV and RVEF (*p* > 0.05). However, the Egger regression test indicates statistically significant bias for RVESV (*p* < 0.05). These results can be observed in the funnel plots in Fig. 3.

According to Table 2, the pooled mean differences for were − 5.064 (95% CI − 10.132, 0.004, *p* > 0.05), 4.654 (95% CI − 4.947, 14.255, *p* > 0.05), − 0.783 (95% CI − 5.630, 4.065, *p* > 0.05, − 0.200 (95% CI − 1.215, 0.815, *p* > 0.05) for LVEF, LVM, RVESV and RVEF, respectively. This indicates no significant difference between RT3DE and CMR for these variables, meaning that results of RT3DE are similar to CMR.

**Table 2:** Mean difference and effect size for all independent variables

Variables	Studies (*n*)	Participants (*n*)	RT3DE		CMR		Mean difference (MD)			Effect size		
			Mean	Relative standard deviation	Mean	Relative standard deviation	MD (95% CI)	*Q* test	*I*^2^ (%)	Effect size (95% CI)	*Q* test	*I*^2^ (%)
Left ventricular end-systolic volume	11	803	75.514	20.813	101.681	22.485	− 16.499 (− 25.297, − 7.702)***	27.219	63.260**	− 0.430 (− 0.640, − 0.221)***	19.543	48.831*
Left ventricular end-diastolic volume	11	803	150.367	9.008	198.837	10.579	− 44.989 (− 65.436, − 24.541)***	80.829	87.628***	− 0.888 (− 1.280, − 0.495)***	63.795	84.325***
Left ventricular ejection fraction	11	803	48.635	8.107	51.421	8.424	− 5.064 (− 10.132, 0.004)	129.028	92.250***	− 0.523 (− 1.009, − 0.038)*	100.973	90.096***
Left ventricular mass	7	324	197.368	12.333	187.680	12.079	4.654 (− 4.947, 14.255)	1.117	0.000	4.654 (− 4.947, 14.255)	1.117	0.000
Right ventricular end-systolic volume	7	518	74.557	13.349	83.976	17.506	− 0.783 (− 5.630, 4.065)	11.379	47.272	0.003 (− 0.244, 0.250)	18.463	67.502**
Right ventricular end-diastolic volume	7	518	132.580	8.432	148.711	12.587	− 5.478 (− 9.334, − 1.622)**	3.546	0.000	− 0.197 (− 0.333, − 0.061)**	3.129	0.000
Right ventricular ejection fraction	7	518	45.763	5.289	45.351	9.668	− 0.200 (− 1.215, 0.815)	5.802	0.000	− 0.005 (− 0.141, 0.131)	5.901	0.000

*p < 0.05, **p < 0.01, ***p < 0.001

Subgroup analyses were conducted for variables with significant heterogeneity, including LVESV, LVEDV, LVEF and RVESV. The subgroup analyses which were conducted indicate some differences which may have contributed to differences in results for each variable as well as significant heterogeneity. For LVESV, there is a significant difference between RT3DE and CMR for those aged over 50 years [MD = − 13.896 (95% CI − 20.480, − 7.311, *p* < 0.001)], but no significant differences for those under 50 [− 21.627 (− 48.932, 5.678)]. Furthermore, studies including only participants with cardiovascular disease indicated a significant mean difference [MD = − 19.286 (95% CI − 33.345, − 5.227), *p* < 0.01] as well as those including both healthy and diseases participants [MD = − 13.657 (95% CI − 23.492, − 3.823), *p* < 0.01]. Additionally, there is a significant difference between RT3DE and CMR for high-quality studies [MD = − 14.784 (95% CI − 21.377, − 8.192), *p* < 0.001], compared to moderate-quality studies indicating no significant difference. Further, studies published in 2010 and earlier also indicated a significant difference between the two diagnostic methods [MD = − 12.301 (95% CI − 20.995, − 3.608), *p* < 0.01] compared to those published after 2010 [MD = − 21.590 (95% CI − 38.495, − 4.685), *p* < 0.05]. Finally, studies using the biplane method indicated a significant difference between RT3DE and CMR [MD = − 14.335 (95% CI − 21.132, − 7.537, *p* < 0.001)] compared to the multiplane method indicating no significant difference [MD = − 7.918 (− 21.416, 5.580), *p* > 0.05)].

For the LVEDV variables, there is a significant difference between RT3DE and CMR for those aged over 50 years [MD = 13.896 (95% CI − 20.480, − 7.311, *p* < 0.001)], but no significant differences for those under 50 [MD = − 52.758 (− 110.466, 4.950), *p* > 0.05]. Furthermore, studies including only participants with cardiovascular disease indicated a significant mean difference [MD = − 19.286 (95% CI − 33.345, − 5.227), *p* < 0.01] as well as those including both healthy and diseases participants [MD = − 13.657 (95% CI − 23.492, − 3.823), *p* < 0.01]. Additionally, there is a significant difference between RT3DE and CMR for high-quality studies [MD = − 14.784 (95% CI − 21.377, − 8.192), *p* < 0.001], compared to moderate-quality studies indicating no significant difference. Further, studies published in 2010 and earlier also indicated a significant difference between the two diagnostic methods [MD = − 12.301 (95% CI − 20.995, − 3.608), *p* < 0.01] as well as those published after 2010 [MD = − 37.027 (95% CI − 66.971, − 7.082), *p* < 0.05]. However the mean difference in studies published before 2010 is more significant. Finally, studies using the biplane method indicated a significant difference between RT3DE and CMR [MD = − 14.335 (95% CI − 21.132, − 7.537, *p* < 0.001)] compared to the multiplane method indicating no significant difference [MD = − 12.958 (− 27.566, 1.649), *p* > 0.05].

For the LVEF variable, for those aged over 50 years, there is a significant mean difference between RT3DE and CMR of − 7.403 (95% CI − 13.852, − 0.954, *p* < 0.05) where RT3DE reports lower left ventricular end-systolic volume compared to those aged 50 years old and under indicating no significant difference [MD = 0.981 (− 7.051, 9.013), *p* > 0.05]. Furthermore, there is no significant mean difference for neither the disease [MD = − 3.002 (− 6.769, 0.764), *p* > 0.05], nor the disease and healthy subgroup [MD = − 8.522 (− 21.784, 4.739), *p* > 0.05]. There is significantly high heterogeneity for mean difference in both studies with diseased participants (*I*^2^ = 91.963, *p* < 0.001) and studies with both diseased and healthy participants (*I*^2^ = 77.387, *p* < 0.001). Additionally, for studies of high quality, RT3DE reports significantly lower LVEDV compared to CMR [MD = − 4.180 (95% CI − 6.882, − 1.478), *p* < 0.01], compared to moderate-quality studies showing no significant difference [− 7.193 (− 26.802, 12.417), *p* > 0.05]. Further, the pooled mean difference for studies published in 2010 and earlier [MD = − 4.159 (95% CI − 6.807, − 1.511), *p* < 0.01], compared to studies published after 2010 indicating no significant difference [MD = − 7.107 (− 21.622, 7.408), *p* > 0.05]. Finally, there is no significant mean difference for neither the biplane [MD = − 9.998 (− 21.211, 1.214), *p* > 0.05] nor the multiplane [MD = 0.944 (− 2.008, 3.896), *p* > 0.05) methods.

For the RVESV variable, there is no significant difference between RT3DE and CMR for mean difference for any subgroups.

Pooled correlation and Bland–Altman analysis results:

According to STable 6*,* there is high correlation between RT3DE and CMR for all variables, and each individual correlation coefficient which was pooled was statistically significant. The statistical analysis method for concordance differed among studies, as specified in Table 1. All variables except for left ventricular end-systolic volume and left ventricular end-diastolic volume have low bias. Although, the limits of agreement for all variables are very wide.

## Discussion

### Summary of overall results

Overall, this meta-analysis indicated no significant mean difference and effect size between RT3DE and CMR for LVEF, LVM, RVESV and RVEF. Additionally, the pooled concordance values and Bland–Altman agreement generated in this meta-analysis for all variables was also very high. This is promising as RT3DE is a cheaper and faster alternative to CMR. Additionally, it provides better quality images to the routinely used two-dimensional echocardiography by removing the need for geometrical assumptions used for the calculation of ventricular mass and volume.

### Similarity between RT3DE and CMR and reason/mechanisms

These similarities can be attributed to the three-dimensional nature of RT3DE which removes spatial and geometric assumption, similar to CMR and therefore produces more accurate ventricular mass and volume calculation. This is different to the standard practice of using two-dimensional echocardiography, subject to inter-observer bias and reduced accuracy due to geometric modelling.

These findings are supported by previous meta-analyses, indicating a low mean difference between RT3DE and CMR with high concordance and low bias within narrow limits of agreement [8–12, 46, 47]. Although according to this meta-analysis, for the variables LVESV, LVEDV and RVEDV, RT3DE reports significantly lower results compared to CMR. In addition, there was significant heterogeneity between studies for the variables LVESV, LVEDV, LVEF and RVESV.

These differences can be attributed to a multitude of reasons related to patient characteristics, study quality and image acquisition and processing method. Therefore in relation to these factors, subgroup analyses, summarised below, were used to identify the sources of heterogeneity and explain any differences between the two methods.

Overall, there is no difference between RT3DE and CMR for the multiplane method, but a significant difference for the biplane method for LVESV and LVEDV. This difference may be because the RT3DE technology was used, rendering the LV volume much smaller than the measured results by CMR. This is consistent with the review result published by Wood et al. [46], suggesting that this negative impact of values in RT3DE relative to the CMR method may have been due to ‘bubble destruction, resulting from the high density of scanlines required for full volumetric acquisition’ [17].

For LVEF, there is no significant difference between RT3DE and CMR for neither the multiplane nor the biplane method. This is consistent with the findings of Yap, van Geuns [12], indicating there is no significant difference between biplane and multiplane methods in LVM determination.

Shimada and Shiota [10] found that the LVM measurement by RT3DE in healthy patients was very accurate in comparison to CMR whereas there was a greater degree of underestimation in patients with cardiovascular diseases. This is similar to the effect size findings of this meta-analysis for LVESV, LVEDV and LVEF variables. We found a moderate (LVESV) or large (LVEDV, LVEF) difference between RT3DE and CMR for the disease subgroup, compared to the diseased and healthy subgroup where there is only a small difference between RT3DE and CMR.

The goal of this subgroup analysis was to determine whether healthy versus diseases heart impacted the difference in results between RT3DE and CMR. Unfortunately, there were insufficient studies on only healthy patients, and therefore studies using a combination of healthy and diseases patients were compared to studies with only patients with cardiovascular disease. However, the small difference between the methodologies for studies with healthy participants, compared to the large differences in studies with only diseases participants, does indicate that diseased hearts may negatively impact RT3DE image quality.

Shimada and Shiota [48] suggest this trend may be due to the lower spatial resolution of RT3DE compared to CMR. In pathologies, dilatation and hypertrophy leads to a great distance between the ultrasound beam and the ventricles, further decreasing image quality. Irregular borders as a result of pathologies, impairing accuracy of RT3DE border tracing and analysis, is suggested to further contribute to greater variation between RT3DE and CMR. This can explain the greater difference between RT3DE and CMR in studies with diseased patients. Based on this, it’s recommended that in future, studies separate participants with cardiovascular disease and healthy participants when analysing data. This can also potentially mitigate the significant heterogeneity for this subgroup statistically indicated in this meta-analysis for the LVESV and LVEDV variables.

Interestingly, this meta-analysis reports no significant mean difference between RT3DE and CMR for moderate-quality studies, however a significant mean difference for high-quality studies for variables LVESV, LVEDV and LVEF. A significant effect size could also be seen in the high-quality study subgroup for each of these variables. However, there is significant heterogeneity in the moderate study quality subgroup. Additionally, since the studies declared to be of high quality by the GRADE assessment tool indicate a significant difference between RT3DE and CMR, then perhaps this significant difference should be considered over the moderate-quality studies. Testing diagnostic methods, particularly imaging, can be difficult due to the expensive and time-consuming nature. Therefore, it is difficult for studies to have a large sample size. If possible, conducting studies with larger sample sizes can increase study quality, which can further validate whether or not there is any significant difference between RT3DE and CMR.

Subgroup analysis was conducted by publication year to determine whether current advancements have made any significant contribution to increasing concordance between RT3DE and CMR. For LVESV and LVEDV, there was a significant difference between CMR and RT3DE for both studies published in 2010 and earlier and those published after 2010. However, for variables LVEDV, studies published in 2010 and earlier had a large effect size, whereas those published after 2010 had only a moderate effect size. This means that more recently published studies indicated a smaller difference between RT3DE and CMR for LVEDV. This is a promising result indicating that recent developments in developments have improved RT3DE imaging analysis and acquisition to increase its concordance with CMR.

Furthermore, for the variables LVEF, where there is a significant effect size and mean difference for studies published in 2010 and earlier, but no significant effect size and mean difference in studies published after 2010. This indicates that RT3DE technology has significantly improved in over the recent years (after 2010), supporting the integration of RT3DE into routine clinical use in the near future.

This meta-analysis reports a significant mean difference and effect size between RT3DE and CMR for LVESV, LVEDV and LVEF for those over the age of 50. However there is no significant difference or effect size between RT3DE and CMR for these variables in those aged 50 years old and under. This may suggest a potential reduced image quality generated by RT3DE in older individuals. Kitzman [49] suggests that due to the normal changes in the heat as a result of age, including increased ventricular wall and valve leaflet thickness, can result in poor-quality images through echocardiography. These findings suggest that potentially, future studies need to differentiate results based on age group as they show different imaging results. This may also help mitigate heterogeneity between studies, which was statistically indicated in this meta-analysis.

Furthermore, aside from age, other biological factors worth further considering, which may potentially impact RT3DE image quality include sex and BMI. However, currently, limited studies can be found focusing or subgrouping by age, sex or BMI [49–51]. Therefore, future studies should also group results based on sex and BMI, in addition to age. The goal, then, is to develop RT3DE to a point where biological differences will not impact image quality.

Therefore, integrating the findings of the subgroup analyses, older patients and the use of the multiplane instead of biplane analysis method may potentially reduce the quality of RT3DE. Additionally, more recent studies indicate no significant difference between RT3DE and CMR, as opposed to older studies, indicating promising development in RT3DE over the past decade [8, 11, 12]. Although considering the significant differences between CMR and RT3DE in terms of heart pathology, older aged patients biplane image analysis methods, further improvement is required in the RT3DE imaging modality prior to integration into routine clinical practice.

## Strengths and limitations

This is the first meta-analysis study which has validated RT3DE against gold standard method, CMR approach including a large number of recently published case–control clinical studies. The meta-analysis has comprehensively assessed the value of RT3DE in clinical application. However, there are a number of limitations in the study. First, the studies used in the meta-analysis themselves had low sample size due to the nature of clinical study. This may explain the significant heterogeneity observed in both variable and subgroup analysis. It is difficult to perform studies in diagnostic methods with large sample sizes and this may have contributed to the lower power and larger margin of error.

Second, there was low agreement between RT3DE and CMR in LV volume assessment, no final judgment can be made about the comparison between RT3DE and CMR in LV volume measurement. A further study encompassing a comparison between RT3DE, CMR and 2D ECHO is needed to confirm the results in our study. Additionally, with improvements to the methodology to increase the agreement between RT3DE and CMR, future studies and meta-analyses are then required to assess similarity.

Furthermore, many studies used a combination of healthy and diseased participants, but did not separate these results. This could have created further variation in results. There were further variations such as difference in equipment, analysis method and participant factors such as ethnicity which subgroup analyses could not be conducted on due to low study number and some studies not reporting on these parameters. Additionally, the number of studies included in the subgroup analyses were also small and therefore may have lacked power to stratify for any methodological differences between the selected studies. Therefore, in future, we aim to reconduct a meta-analysis once more studies have been published in the field, to produce a meta-analyses with a higher power. In addition, we hope to potentially find more studies which include a larger sample size.

## Conclusion

This meta-analysis included a very detailed analysis in terms of difference between RT3DE and CMR. Further steps have been taken in subgroup analyses which previous studies have not conducted. Through the collation of data from a range of different countries and RT3DE methods, the generalisability of study findings are high.

Overall, this meta-analysis indicated promising results for the use of RT3DE, with no significant difference to CMR (for LVEF, RVESV and RVEF). Although in some cases, RT3DE appears to underestimate volumes when compared to CMR (LVESV, LVEDV and RVEDV).

Currently, the most commonly used analysis method is semi-automatic where the borders are manually traced by the observer. Although, improvements are still being made to fully automate the process, to reduce processing time. In addition, currently analysis is conducted via a biplane a multiplane method. This meta-analysis indicates that there is no difference between RT3DE and CMR for the multiplane method, but a significant difference for the biplane method for LVESV and LVEDV. This indicates that the multiplane method is potentially a superior method. This is supported by previous research suggesting the benefit of the multiplane method in more in-depth analysis of heart structures, the results surrounding this is inconclusive and further research and development is required here.

Further advancements are required to compensate for biological changes including age, sex and BMI. This is of particular importance considering that the demographics most in need of these imaging methods are patients over the age of 50 with heart pathologies. This meta-analysis indicates lower concordance between RT3DE and CMR for older individuals. There is also greater underestimation by RT3DE compared to CMR in individuals with diseased heart, as indicated in this study. Previous research has suggested that RT3DE had provide more detail into heart structures when compared with the routinely used 2DE method. However, the above developments are being made to improve temporal and spatial resolution, which is lower in RT3DE compared to 2DE.

With technological advancement, RT3DE can be integrated into routine clinical practice. Further development should improve efficiency, workflow, image quality, speed, accuracy and simplicity of the RT3DE method. This will make RT3DE more accessible, and likely to be chosen over the current, more expensive and time-consuming method of CMR.

### Supplementary Information

Below is the link to the electronic supplementary material.

Supplementary file1 (DOCX 38 kb)

## Data Availability

Data will be available upon request.
