# Supplementary material for: Validating real-time three-dimensional echocardiography against cardiac magnetic resonance, for the determination of ventricular mass, volume and ejection fraction: a meta-analysis
Source: Clin Res Cardiol. 2023 Apr 20;113(3):367–92. doi: 10.1007/s00392-023-02204-5 (PMC10881629; doi:10.1007/s00392-023-02204-5)
Supplement: Supplementary file 1 — Supplementary file1 (DOCX 38 kb) [file 392_2023_2204_MOESM1_ESM.docx]

| Mean Difference | | | | | | Effect Size | | |
| --- | --- | --- | --- | --- | --- | --- | --- | --- |
| Subgroups | Studies (n) | Participants (n) | MD (95% CI) | Q test | I^2^ (%) | Effect Size (95% CI) | Q test | I^2^ (%) |
| Age |  |  |  |  |  |  |  |  |
| 50 years old and under | 3 | 185 | -21.627 (-48.932, 5.678) | 21.130 | 90.535*** | -0.670 (-404, 0.064) | 8.989 | 77.749* |
| More than 50 years old | 8 | 258 | -13.896 (-20.480, -7.311)*** | 6.030 | 0.000 | -0.329 (-0.490, -0.167)*** | 6.121 | 0.000 |
| Disease |  |  |  |  |  |  |  |  |
| Disease | 6 | 138 | -19.286 (-33.345, -5.227)** | 26.236 | 80.942*** | -0.649 (-1.015, -0.283)** | 10.965 | 54.399 |
| Disease and Healthy | 5 | 234 | -13.657 (-23.492, -3.823)** | 0.978 | 0.000 | -0.251 (-0.433, -0.069)** | 0.993 | 0.000 |
| Study Quality |  |  |  |  |  |  |  |  |
| High | 7 | 235 | -14.784 (-21.377, -8.192)*** | 5.372 | 0.000 | -0.389 (-0.572, -0.206)*** | 5.921 | 0.000 |
| Moderate | 4 | 96 | -18.051 (-41.221, 5.120) | 21.425 | 85.998*** | -0.434 (-0.958, 0.090) | 13.560 | 77.876** |
| Publication Year |  |  |  |  |  |  |  |  |
| 2010 and earlier | 6 | 161 | -12.301 (-20.995, -3.608)** | 10.750 | 50.240 | -0.447 (-0.689, -0.204)*** | 6.000 | 12.850 |
| After 2010 | 5 | 211 | -21.590 (-38.495, -4.685)* | 11.800 | 64.510* | -0.418 (-0.792, -0.044)* | 13.310 | 72.77* |
| RT3DE Analysis Planes |  |  |  |  |  |  |  |  |
| Biplane | 6 | 165 | -14.335 (-21.132, -7.537)*** | 5.950 | 0.000 | -0.458 (-0.709, -0.206)*** | 6.030 | 21.630 |
| Multiplane | 3 | 109 | -7.918 (-21.416, 5.580) | 3.080 | 40.290 | -0.234 (-0.478, 0.0100) | 0.700 | 0.000 |

Supplemental STable 1: Left ventricular end-systolic volume (LVESV) subgroup analysis

*p<0.05, **p<0.01, ***p<0.001

| Mean Difference | | | | | | Effect Size | | |
| --- | --- | --- | --- | --- | --- | --- | --- | --- |
| Subgroups | Studies (n) | Participants (n) | MD (95% CI) | Q test | I^2^ (%) | Effect Size (95% CI) | Q test | I^2^ (%) |
| Age |  |  |  |  |  |  |  |  |
| 50 years old and under | 3 | 185 | -52.758 (-110.466, 4.950) | 42.542 | 95.299*** | -1.182 (-2.277, -0.087)* | 17.635 | 88.659*** |
| More than 50 years old | 8 | 258 | -41.736 (-62.692, -20.780)*** | 38.280 | 81.714*** | -0.772 (-1.170, -0.375)*** | 37.529 | 81.348*** |
| Disease |  |  |  |  |  |  |  |  |
| Disease | 6 | 138 | -64.572 (-98.740, -30.403)*** | 62.211 | 91.963*** | -1.382 (-1.949, -0.815)*** | 22.111 | 77.387*** |
| Disease and Healthy | 5 | 234 | -22.846 (-35.293, -10.399)*** | 5.252 | 23.846 | -0.378 (-0.589, -0.166)*** | 5.293 | 24.427 |
| Study Quality |  |  |  |  |  |  |  |  |
| High | 7 | 235 | -53.712 (-74.810, -32.615)*** | 27.268 | 77.996*** | -1.056 (-1.537, -0.575)*** | 34.397 | 82.557*** |
| Moderate | 4 | 96 | -29.711 (-68.790, 9.367) | 40.660 | 92.622*** | -0.602 (-1.354, 0.151) | 26.831 | 88.819*** |
| Publication Year |  |  |  |  |  |  |  |  |
| 2010 and earlier | 6 | 161 | -52.241 (-83.103, -21.379)** | 50.600 | 90.600*** | -1.118 (-1.720, -0.515)*** | 31.050 | 83.010*** |
| After 2010 | 5 | 211 | -37.027 (-66.971, -7.082)* | 1.300 | 0.000 | -0.652 (-1.237, -0.067)* | 27.990 | 88.390*** |
| RT3DE Analysis Planes |  |  |  |  |  |  |  |  |
| Biplane | 6 | 165 | -52.951 (-83.189, -22.712)** | 35.830 | 87.880*** | -1.105 (-1.723, -0.487)*** | 35.940 | 84.950*** |
| Multiplane | 3 | 109 | -12.958 (-27.566, 1.649) | 2.520 | 30.000 | -0.299 ( -0.543, -0.054)** | 0.610 | 0.000 |

Supplemental STable 2: Left ventricular end-diastolic volume (LVEDV) subgroup analysis.

*p<0.05, **p<0.01, ***p<0.001

*Supplemental STable 3: Left ventricular ejection fraction (LVEF) subgroup analysis.*

| Mean Difference | | | | | | Effect Size | | |
| --- | --- | --- | --- | --- | --- | --- | --- | --- |
| Subgroups | Studies (n) | Participants (n) | MD (95% CI) | Q test | I^2^ (%) | Effect Size (95% CI) | Q test | I^2^ (%) |
| Age |  |  |  |  |  |  |  |  |
| 50 years old and under | 3 | 185 | 0.981 (-7.051, 9.013) | 14.415 | 86.126** | -0.076 (-0.987, 0.835) | 14.193 | 85.909** |
| More than 50 years old | 8 | 258 | -7.403 (-13.852, -0.954)* | 107.117 | 93.465*** | -0.686 (-1.261, -0.110)* | 78.805 | 91.117*** |
| Disease |  |  |  |  |  |  |  |  |
| Disease | 6 | 138 | -3.002 (-6.769, 0.764) | 24.655 | 79.720*** | -0.543 (-1.217, 0.131) | 36.531 | 86.313*** |
| Disease and Healthy | 5 | 234 | -8.522 (-21.784, 4.739) | 104.114 | 96.158*** | -0.505 (-1.269, 0.259) | 64.009 | 93.751*** |
| Study Quality |  |  |  |  |  |  |  |  |
| High | 7 | 235 | -4.180 (-6.882, -1.478)** | 17.778 | 66.250** | -0.571 (-1.007, -0.136)* | 30.548 | 80.358*** |
| Moderate | 4 | 96 | -7.193 (-26.802, 12.417) | 111.068 | 97.299*** | -0.406 (-1.654, 0.843) | 70.063 | 95.718*** |
| Publication Year |  |  |  |  |  |  |  |  |
| 2010 and earlier | 6 | 161 | -4.159 (-6.807, -1.511)** | 12.621 | 60.382* | -0.623 (-1.157, -0.088)* | 25.590 | 80.461*** |
| After 2010 | 5 | 170 | -7.107 (-21.622, 7.408) | 116.378 | 96.563*** | -0.405 (-1.275, 0.466) | 74.016 | 94.596*** |
| RT3DE Analysis Planes |  |  |  |  |  |  |  |  |
| Biplane | 6 | 165 | -9.998 (-21.211, 1.214) | 87.480 | 97.710*** | -1.043 (-1.719, -0.366) | 48.910 | 87.380*** |
| Multiplane | 3 | 109 | 0.944 (-2.008, 3.896) | 0.170 | 0.000 | 0.085 (-0.158, 0.328) | 0.090 | 0.000 |

*p<0.05, **p<0.01, ***p<0.001

| Mean Difference | | | | | | Effect Size | | |
| --- | --- | --- | --- | --- | --- | --- | --- | --- |
| Subgroups | Studies (n) | Participants (n) | MD (95% CI) | Q test | I^2^ (%) | Effect Size (95% CI) | Q test | I^2^ (%) |
| Age |  |  |  |  |  |  |  |  |
| 50 years old and under | 3 | 220 | 0.617 (-6.949, 8.183) | 4.440 | 54.870 | 0.129 (-0.374, 0.631) | 14.400 | 85.810** |
| More than 50 years old | 4 | 199 | -3.253 (-8.621, 2.116) | 0.550 | 0.000 | -0.116 (-0.314, 0.081) | 0.620 | 0.000 |
| Study Quality |  |  |  |  |  |  |  |  |
| High | 3 | 209 | -3.432 (-8.956, 2.092) | 0.620 | 0.000 | -0.119 (-0.311, 0.074) | 0.620 | 0.000 |
| Moderate | 4 | 210 | 0.845 (-5.772, 7.461) | 4.460 | 41.300 | 0.091 (-0.321, 0.502) | 13.910 | 74.920** |
| Publication year |  |  |  |  |  |  |  |  |
| 2010 and earlier | 3 | 248 | -0.052 (-7.029, 6.924) | 8.010 | 71.340* | 0.110 (-0.403, 0.624) | 16.340 | 88.020*** |
| After 2010 | 4 | 171 | -2.680 (-9.733, 4.373) | 0.540 | 0.000 | -0.091 (-0.304, 0.122) | 0.390 | 0.000 |

Supplemental STable 4: Right ventricular end-systolic volume subgroup analysis.

*p<0.05, **p<0.01, ***p<0.001

Supplemental STable 5: Egger regression results for publication bias (two-tailed p-value).

| **Variable** | **Egger test** | **P value** |
| --- | --- | --- |
| **Left ventricular end-systolic volume** | -2.815 (-6.001, 0.371) | 0.077 |
| **Left ventricular end-diastolic volume** | -6.463 (-14.075, 1.150) | 0.087 |
| **Left ventricular ejection fraction** | -0.341 (-9.544, 8.863) | 0.935 |
| **Left ventricular mass** | -0.805 (-1.620, 0.010) | 0.052 |
| **Right ventricular end-systolic volume** | -1.675 (-2.727, -0.624) | 0.009 |
| **Right ventricular end-diastolic volume** | -0.588 (-1.944, 0.767) | 0.315 |
| **Right ventricular ejection fraction** | 0.040 (-2.429, 2.350) | 0.968 |

STable 6: Pooled correlation and Bland-Altman (bias and limits of agreement [LOA]) results.

| **Variable** | **Correlation coefficient** | **Bland-Altman bias** | **Bland-Altman upper LOA** | **Bland-Altman lower LOA** |
| --- | --- | --- | --- | --- |
| **Left ventricular end-systolic volume** | 0.907 | -77.800 | 313.368 | -504.708 |
| **Left ventricular end-diastolic volume** | 0.896 | -15.423333 | 35.6406667 | -60.519 |
| **Left ventricular ejection fraction** | 0.860 | -0.8341667 | 14.6043333 | -9.071 |
| **Left ventricular mass** | 0.880 | 1.504 | 59.741 | -56.788 |
| **Right ventricular end-systolic volume** | 0.848 | -1.638 | 35.282 | -38.775 |
| **Right ventricular end-diastolic volume** | 0.860 | -3.927 | 45.908 | -53.757 |
| **Right ventricular ejection fraction** | 0.734 | -0.504 | 17.086 | -18.094 |
